# Macphail’s Null Hypothesis of Vertebrate Intelligence: Insights From Avian Cognition

**DOI:** 10.3389/fpsyg.2020.01692

**Published:** 2020-07-08

**Authors:** Amalia P. M. Bastos, Alex H. Taylor

**Affiliations:** School of Psychology, The University of Auckland, Auckland, New Zealand

**Keywords:** null hypothesis, avian cognition, comparative psychology, differences in intelligence, avian intelligence

## Abstract

Macphail famously criticized two foundational assumptions that underlie the evolutionary approach to comparative psychology: that there are differences in intelligence across species, and that intelligent behavior in animals is based on more than associative learning. Here, we provide evidence from recent work in avian cognition that supports both these assumptions: intelligence across species varies, and animals can perform intelligent behaviors that are not guided solely by associative learning mechanisms. Finally, we reflect on the limitations of comparative psychology that led to Macphail’s claims and suggest strategies researchers can use to make more advances in the field.

## Introduction

Euan Macphail sparked great controversy in the 1980s, following his synthesis of the current state of comparative psychology. Macphail argued that, given the body of evidence available at the time, there appeared to be no quantitative or qualitative differences in intelligence across species (Macphail, 1982, 1985; Macphail and Bolhuis, 2001), and that seemingly intelligent behavior is underpinned by associative learning (Macphail, 1982, 1985; Macphail and Bolhuis, 2001). In Macphail’s general process view, species differences in performance within tasks could be ascribed to contextual variables, rather than to any real differences in their underlying cognition. He directly pitted this view against the widely-regarded ecological view (Macphail and Bolhuis, 2001), which takes an evolutionary perspective of cognition, suggesting that species evolve cognitive adaptations to their environment, just as they do physiological adaptations. Here, we will consider two of these lines of Macphail’s criticism in light of recent developments in avian cognition: namely, that there are differences in intelligence across species, and that intelligent behavior cannot be explained by associative learning alone.

## Differences in Intelligence Across Species

Macphail’s null hypothesis of vertebrate intelligence posits that all animals use the same general mechanisms, to the same level of ability, to solve cognitive tasks. Whilst this may be true in considering some basic processes such as operant conditioning, which appear to be universal across species, this hypothesis fails to consider differences in intelligence at finer, and more ecologically relevant, scales (Shettleworth, 1987).

A strong line of evidence suggesting not only that intelligence is quantitatively different across species, but that these differences emerge as a direct consequence of their ecology, explores the relationship between species’ social complexity and cognitive task performance. The social intelligence hypothesis predicts that complex social environments require better memory and overall cognitive capacity, and so social complexity drives the evolution of intelligence (Dunbar, 1998, 2008). Comparative studies across both parrot and corvid species provide support for this hypothesis. For example, parrots living in complex groups involving fission-fusion dynamics outperform those that form smaller and more stable family groups in string-pulling tasks testing means-end comprehension (Krasheninnikova et al., 2013). Similarly, pinyon jays, which live in large flocks of up to five hundred individuals, outperform western scrub-jays, which form small family groups, in tasks of transitive inference (Bond et al., 2003). Pinyon jays also outperform two less social species, western scrub-jays and Clark’s nutcrackers, in both color and spatial reversal tasks (Bond et al., 2007). Correlations between social complexity and cognitive capacity may be particularly strong in the corvids and parrots, due to the long life expectancy in these species, which might facilitate exposure to a greater number of social partners over time (Boucherie et al., 2019).

There is also good evidence that quantitative cognitive differences between species are driven by their ecological differences in comparative work between caching and non-caching corvids. While caching and non-caching corvids perform, similarly, in a color-based task, caching species outperform non-caching species in a spatial task (Olson et al., 1995). Findings from studies such as these suggest that ecology plays an important role in shaping the cognitive abilities of species. Given that comparative psychology has so far been restricted to a minority of species, it seems likely that as a greater number of species are tested, more differences in intelligence are likely to emerge (Elepfandt, 1987; Kamil, 1987; Shultz and Dunbar, 2010; van Horik and Emery, 2011), generating more clear and testable links between differences in ecology and cognitive ability.

Evidence for convergent evolution in the cognitive abilities of great apes, corvids, and parrots also suggests that quantitative differences in intelligence do exist across species, and that these differences relate to their ecology (Emery, 2004; Clayton, 2012; Emery et al., 2012; Güntürkün and Bugnyar, 2016; Auersperg and von Bayern, 2019). One clear prediction that the contextual variable argument makes is that differences in methodology should become more difficult to control for and, therefore, have a greater impact on task performance, the further apart two species are phylogenetically. This is because species that are more similar are more likely to share more of the same perceptual abilities and biases than those that are more distantly related. Thus, if species do not differ in intelligence, as Macphail claims, we should expect problem solving performances to differ more as phylogenetic distance increases, due to contextual variables becoming harder to control.

However, the great apes, parrots, and corvids, despite being evolutionarily distant taxa, converge in several of their cognitive abilities (Emery, 2004, 2006; Emery and Clayton, 2004; Seed et al., 2009; Clayton, 2012; Emery et al., 2012; Güntürkün and Bugnyar, 2016; Auersperg and von Bayern, 2019). The Piagetian framework for object permanence describes different stages of development for this ability, which requires an individual to understand that an object continues to exist when hidden within a container (Piaget, 1954). Its final stage requires an understanding of invisible displacement: that is, tracking a container which presumably contains the hidden object as it moves behind a series of screens or occluders, and guessing where it may have been left once the container is shown to be empty. The great apes (de Blois et al., 1998; Call, 2001; Collier-Baker and Suddendorf, 2006; Collier-Baker et al., 2006; Mallavarapu, 2009), corvids (Pollok et al., 2000; Zucca et al., 2007; Hoffmann et al., 2011; Ujfalussy et al., 2013), and parrots (Pepperberg and Funk, 1990; Pollok et al., 2000) succeed at the final stage of object permanence, even though other species of both birds and mammals do not. Four species of lemurs (Deppe et al., 2009; Mallavarapu, 2009) succeed only at visible displacement tasks, where the reward can be seen as it moves between two or more occluders. Several other mammals also fail to understand invisible displacement tasks (for a review, see Jaakkola, 2014), even though they understand visible displacements, suggesting that contextual variables are not to blame. Similarly, ring doves can successfully retrieve a hidden reward, but fail to track its displacement within a container (Dumas and Wilkie, 1995). Given that parrots, corvids, and the great apes show similar performance whilst more closely related mammalian and avian species fail, it seems likely that stage 6 object permanence – the ability to understand invisible displacements–emerged convergently in the great apes and these two avian taxa, and represents a real quantitative difference in cognitive ability across species.

A similar convergence in capacity appears in the object transposition task. In this task, a reward is hidden under one of two cups, and their positions are changed. In children, the ability to solve the transposition task emerges later than the ability to solve invisible displacement tasks (Sophian and Sage, 1983; Sophian, 1984; Barth and Call, 2006), suggesting that this is a more challenging type of problem. A large number of mammals either fail to solve transposition tasks or may use associative strategies to guide their choices, including cats (Doré et al., 1996), dogs (Doré et al., 1996; Rooijakkers et al., 2009; Fiset and Plourde, 2013), wolves (Fiset and Plourde, 2013), wild boars (Albiach-Serrano et al., 2012), pigs (Albiach-Serrano et al., 2012), goats (Nawroth et al., 2015), dolphins (Jaakkola et al., 2010), and bears (Hartmann et al., 2017). Despite this selection of species including herbivores, omnivores, and carnivores, as well both domesticated and wild animals, only parrots (Pepperberg et al., 1997; Auersperg et al., 2014) and primates (Beran and Minahan, 2000; Call, 2001, 2003; Beran et al., 2005; Barth and Call, 2006; Rooijakkers et al., 2009) have been conclusively shown to succeed at object transposition tasks. Rather than relying on associative learning strategies, these two taxa appear able to represent the change to the objects’ spatial locations.

Another example is the ability to reason through inference by exclusion (Güntürkün and Bugnyar, 2016). In tests of inference by exclusion, subjects must infer that one of two stimuli contains or is associated with a reward, after a demonstration that the other stimulus is not. Where two cups are used, for example, they must reason that if the reward is not hidden in the cup shown to be empty, then it must be in the other one. Several species of corvids (Schloegl et al., 2009; Mikolasch et al., 2012; Shaw et al., 2013; Jelbert et al., 2015), parrots (Schloegl et al., 2009; Mikolasch et al., 2011; Pepperberg et al., 2013; O’Hara et al., 2015, 2016; Bastos and Taylor, 2019; Subias et al., 2019), and apes (Call, 2004, 2006; Hill et al., 2011) readily reason in this way. The ability to reason by exclusion is present in some New World monkeys (Sabbatini and Visalberghi, 2008; Marsh et al., 2015; Takahashi et al., 2015). Some capuchin monkeys are capable of this form of inference, whilst squirrel monkeys fail at both auditory and visual versions of the task (Marsh et al., 2015). This ability seems to be absent from other mammalian species including rats, golden hamsters, and tree shrews (Takahashi et al., 2015). This pattern suggests that the ability to reason through inference by exclusion varies quantitatively across species and has emerged convergently in the primate and avian lineages.

The ability to reason about use probabilistic information to make predictions about uncertain events also appears to have evolved convergently in the great apes and parrots (Rakoczy et al., 2014; Bastos and Taylor, 2020). When choosing between two hidden samples taken from two mixed populations of rewarding and unrewarding objects, capuchin monkeys appear to use a heuristic strategy of simply avoiding the sample from the population with the greatest absolute number of unrewarding objects (Tecwyn et al., 2017). On the other hand, both the great apes and the New Zealand mountain parrot, the kea, make their choices by relying on probabilistic information, by comparing the ratios of objects within and between the two populations (Rakoczy et al., 2014; Bastos and Taylor, 2020). This ability, known as true statistical inference, has so far not been conclusively shown outside of these two taxa, as other studies on primates and birds have not been able to exclude the absolute number heuristic as a potential strategy (Clements et al., 2018; Roberts et al., 2018; De Petrillo and Rosati, 2019; Placì et al., 2019).

Macphail went further than suggesting there are no quantitative differences in intelligence between species. He also suggested there are no qualitative differences in intelligence across species (Macphail, 1982, 1985; Macphail and Bolhuis, 2001). Research in this area has rather focused on whether there are types of thought that are unique to humans (Penn et al., 2008), rather than whether different animal species might think in qualitatively different ways. At present, therefore, it is not clear whether this hypothesis has been tested sufficiently to make conclusions either way. One route to testing this hypothesis further is focusing more on testing whether there are differences in the information processing biases, errors and limits of species showing similar levels of performance at different behavioral tasks (Taylor, 2014).

## Intelligent Behavior Beyond Pure Association

Another of Macphail’s claims is that all intelligent behavior can be explained by associative learning alone (Macphail, 1982, 1985; Macphail and Bolhuis, 2001). However, critics of Macphail have highlighted that a purely associative view of cognition is insufficient to explain the intelligent behaviors observed in vertebrates (Shettleworth, 1987), including birds. There are certainly areas of the literature on avian cognition where there is great debate as to whether the problem solving performances of birds can be explained by associative learning alone. For example, there is currently debate surrounding the role of associative learning and more complex cognition in research on planning in ravens (Redshaw et al., 2017; Lind, 2018; Dickerson et al., 2018; Hampton and Hampton, 2019), stone-dropping in corvids (Taylor and Gray, 2009; Cheke et al., 2011; Taylor et al., 2011; Jelbert et al., 2014; Logan et al., 2014; Ghirlanda and Lind, 2017; Hennefield et al., 2018, 2019), and string-pulling in a wide variety of birds (Taylor et al., 2010b, 2012; for a review of the species tested on string pulling, see Jacobs and Osvath, 2015). However, several lines of evidence indicate the presence of specific cognitive mechanisms other than associative learning in birds.

First, work on the innate cognitive capacities of birds has shown that prior experience is not required for complex problem solving to emerge. Without any prior experience, chicks can solve several problems in the physical realm, including detouring around a barrier by moving away from a desired object (Regolin et al., 1995), mentally representing the possible location of a hidden object when choosing between two different screens (Vallortigara et al., 1998; Chiandetti and Vallortigara, 2011), and recognizing partially-hidden objects by representing their complete outline (Regolin and Vallortigara, 1995; Regolin et al., 2004). Research on imprinted ducklings has also revealed an innate ability to distinguish between the abstract concepts of “same” and “different”: when imprinted on two identical objects, ducklings preferred to approach pairs of identical objects rather than pairs of different objects, even though the objects in either case were different from those they were originally imprinted on (Martinho and Kacelnik, 2016). Given that these studies used inexperienced chicks and ducklings, this line of work strongly suggests that intelligence operates on more cognitive processes than associative learning alone. Work in chicks also offers further support for an innate approximate number system. Inexperienced chicks can distinguish between both small quantities from one to four (Rugani et al., 2013a) and larger quantities between five and ten (Rugani et al., 2013b). This capacity develops in birds into a numerical ability of surprising complexity. A seminal study in pigeons trained subjects to select images including one, two, or three shapes in ascending order, after which pigeons were asked to order sets with numerosities between one and nine (Scarf et al., 2011). Pigeons succeeded in this task despite never having received training on stimuli including between four and nine shapes, suggesting that they represent one through nine on an ordinal scale.

Work on the social cognition of birds has found clear evidence of birds performing beyond the predictions of associative learning. In a recent prosociality experiment, African gray parrots readily transferred tokens through a window to a conspecific who could exchange them for a food reward, when they could not exchange them themselves (Brucks and von Bayern, 2020). The study’s control conditions suggest that this response was not driven by associative learning alone, as token transfers occurred significantly less often when their partner was unable to exchange tokens, or when the partner was absent. Similarly, an associative account would suggest that their tendency to transfer tokens would increase over time, but most subjects acted prosocially in their first trial. Caching studies provide evidence that birds can flexibly use information learnt in an egocentric manner to make allocentric predictions about the behavior of conspecifics in their environment. For example, Western scrub-jays, which pilfer other individual’s caches, strategically relocate their caches (Emery and Clayton, 2001; Dally et al., 2005, 2006) in response to novel cues of a conspecific’s presence, so as to reduce the likelihood of their caches being stolen in the future. In order to do this, individuals must have pilfered others’ caches before, but need not have observed a pilfering event by another individual (Emery and Clayton, 2001), suggesting that they can project their own experience onto others. An associative learning explanation fails to acknowledge how they might shift between these egocentric and allocentric perspectives. A more recent study on ravens shows these birds will re-cache food when they believe they are being watched, and not as a learned response to a conspecific’s gaze (Bugnyar et al., 2016). Ravens were similarly fast to cache when they heard sound recordings of a conspecific in a nearby compartment with a peephole, which could have granted the conspecific visual access to the cache, and when a conspecific was fully visible in the nearby compartment. In contrast, ravens cached slower and made more improvements to their caches in a control non-observed condition where they could hear a conspecific in a nearby compartment, but this conspecific was neither visible nor had a peephole available to look through. Ravens, therefore, appeared to flexibly use their egocentric experiences, in this case looking through a peephole at the caching chamber, to predict that another individual at the peephole would be able to see them caching.

Work on tool use in birds have produced a number of intriguing findings, suggesting that birds are capable of sophisticated technical intelligence (Weir et al., 2002; Taylor et al., 2007, 2010a; Tebbich et al., 2007; Bird and Emery, 2009; von Bayern et al., 2009, 2018; Wimpenny et al., 2009; Auersperg et al., 2010, 2011b, 2012b; Teschke and Tebbich, 2011; St Clair and Rutz, 2013; Laumer et al., 2016; Jelbert et al., 2018, 2019; Fayet et al., 2020). While some of these studies suggested that birds might be capable of mental trial and error during tool use, conclusive evidence that birds can mentally represent tool problems only emerged recently, from a study on New Caledonian crows (Gruber et al., 2019). This showed that these birds can pre-plan a sequence of behaviors up to three steps ahead, taking an available tool to the correct apparatus (the sub-goal) in order to retrieve another tool, which only then could be used to obtain a food reward (the overall goal). New Caledonian crows correctly planned and executed this sequence of behaviors even though all components of the sequence were out-of-sight of each other. This, therefore, required the crows to mentally represent the location and identity of the correct out-of-sight sub-goal and then use this representation to from a plan to solve the problem without error. Clear evidence of future-directed thought also comes from work on caching corvids. Western scrub-jays can anticipate their future needs, storing food that is unlikely to be available the following morning in a particular location (Alexis et al., 2007; Cheke and Clayton, 2011), regardless of their current satiation state (Correia et al., 2007). Evidence for the use of mental representations during tool manufacture has also emerged recently. After learning to insert a tool of a particular size into a vending machine, New Caledonian crows, when given a sheet of paper, were able manufacture tools of the correct size to insert into the machine (Jelbert et al., 2018). This was despite no tool template being available at the time of manufacture for use as a reference. Instead, the crows had to rely solely on their mental representation of the tool’s size. Additionally, crows were not rewarded at test for making tools of the correct size. Instead, half of all tools made were rewarded irrespective of size, meaning there was no differential reinforcement for making the correct size tool at test.

Recently, work has begun to show that birds can solve problems that require domain-general intelligence, rather than problems involving domain-specific, ecologically relevant behaviors such as tool use and caching. Initial evidence that birds might have more domain-general cognitive processes comes from studies examining the ability of non-tool users to solve tool problems (Bird et al., 2009; Auersperg et al., 2010, 2011a,b, 2012b, 2016; Laumer et al., 2016). More recently, a study in kea showed that they can not only make accurate probabilistic comparisons between the two sampling events, as described above, but also integrate information across different domains (Bastos and Taylor, 2020). In one experiment, the two jars contained a physical barrier, and the otherwise identical populations of tokens were unevenly distributed above and below these barriers. Kea considered the physical constraint imposed by the barriers, adjusting their predictions of the likely sampling outcomes from the two jars. Another experiment in this study provided the kea with social information on sampling biases: one human demonstrators showed they had a preference for rewarding tokens by taking them from a jar rewarding tokens were in the minority, while the other demonstrated they were an unbiased blind sampler by taking rewarding tokens from a jar where such tokens were in the majority. When both these demonstrators sampled from jars with an even split of rewarding and unrewarding tokens, kea preferentially selected the samples from the biased demonstrator. These results showed that kea integrated either social or physical information into their probabilistic predictions, performing comparably to human infants (Téglás et al., 2007; Xu and Denison, 2009; Denison and Xu, 2010, 2014; Denison et al., 2012) and chimpanzees (Eckert et al., 2018a, b; Rakoczy et al., 2014), and outperforming monkeys (Tecwyn et al., 2017).

## Echoes of Macphail’s Criticisms in the 21st Century

Despite recent research not finding support for several of Macphail’s claims, it is important to consider why Macphail may have raised these points in the first place, and why they are relevant today. The reasoning behind Macphail’s null hypothesis for differences in intelligence across vertebrates highlights a flaw that has pervaded comparative psychology for many years: it is often impossible to tell why animals fail at a task. Differences in apparatus, methodology, motivation, and other contextual factors may affect species’ performance in cognitive tasks. As highlighted by Macphail, failure at a task might be a true reflection of the species’ ability, or the result may be caused by some contextual variable in that task. Researchers may attempt to resolve this in two ways: either by presenting an identical task across species, or by modifying some contextual variables in the task so it is better suited to a particular species. However, these two solutions are equally problematic.

When contextual variables are changed to suit a particular species, this makes it even more difficult to establish the reason for a species’ failure at the task (Caldwell and Whiten, 2002; Schloegl et al., 2009; Liedtke et al., 2011; Auersperg et al., 2012a; Krasheninnikova et al., 2019; Farrar et al., 2020). Small changes in contextual variables may affect how a species interprets a task and therefore affect their performance, and this is likely to make it difficult to compare performances in a task across multiple species. One clear example of this comes from work on the trap-tube, a problem where an animal must extract food from a tube with a tool while avoiding a trap set into the lower surface of the tube. Apes’ performance at this task changed dramatically once subjects were allowed to pull food with a tool toward them, rather than push food away (Mulcahy and Call, 2006), with learning speed increasing greatly and subjects passing the key “inverted-tube” control, where the tube was turned upside down, rendering the trap irrelevant. This example highlights how small changes to a task can affect animal performance greatly and offers a cautionary reminder of how hard it can be to interpret failure by a species at a cognitive task.

Even presenting an identical task to two very different species may lead to false positives, or false negatives, when the two species interpret the same task differently. This has been highlighted in a number of studies where animals failed at tasks involving a human demonstrator, but could have performed better had that contextual variable been changed (Erdőhegyi et al., 2007; Mikolasch et al., 2012; Shaw et al., 2013; Nawroth et al., 2014; Jelbert et al., 2016). Given that failure at a task does not necessarily represent a species’ true cognitive abilities, negative results often become ambiguous and difficult to interpret, contributing to a “file-drawer effect” and publication bias (Fanelli and Fanelli, 2012; Farrar and Ostojić, 2019).

These issues in comparative psychology are highlighted in a landmark study by Maclean and colleagues (MacLean et al., 2014), which presented two identical tasks across thirty-six species to measure self-control: an A-not-B task, where a reward was visibly moved between two locations after being previously rewarded in only one of them, and a cylinder task, where an opaque cylinder containing food was presented and then substituted for a translucent cylinder. According to the authors, greater self-control should enable species to successfully switch search locations in the A-not-B task and avoid reaching directly for the food in the cylinder task. However, the study failed to consider how different species may perceive these tasks (Jelbert et al., 2016; Kabadayi et al., 2017): for example, that birds may perform poorly in the A-not-B task due to a poor innate understanding of human hands (Jelbert et al., 2016), rather than an inability to exert self-control. In support of this critique, New Caledonian crow performed poorly at this task without experience tracking hands, but after hand-tracking training actually performed comparably to the great apes in the same task (Jelbert et al., 2016).

Macphail’s view suggests that errors such as these could be ruled out by exhaustively varying perceptual task features and other contextual variables to ensure that they are not responsible for subjects’ failures, but in real terms this is often impossible (Kamil, 1987). One potential solution to this problem is to present pre-test baselines to different species (Jelbert et al., 2016). These baselines would comprise simple tasks that the animal would be expected to succeed at, given that the testing methods – or contextual variables – were appropriate. Success at such baselines could act as a checkpoint prior to test, ensuring that all species in the experiment understood the basic requirements of the test. Provided that a species succeeded at these baseline tasks, it would be possible to more confidently attribute failure at test to a lack of understanding, rather than other aspects of the task. For example, in the New Caledonian crow A-not-B study mentioned above, subjects first experienced hand-tracking training, watching the experimenter’s hand bait a container among multiple other hand movements involving several cups and their lids (Jelbert et al., 2016). Had the subjects not first experienced this baseline training, it would not have been possible to determine if failure at the subsequent A-not-B task was due to a lack of experience with tracking human hands, or reflected a failure to inhibit a response to investigate a previously-rewarded container. Similarly, as highlighted earlier, the performance of various mammal species that pass visible displacement tasks, but fail invisible displacement tasks, provides stronger evidence for this failure being due to cognition rather than contextual variables, because the visible displacement task acts as a baseline test for the more complex invisible displacement task.

Another criticism of MacLean et al. (2014) is a lack of clarity on exactly which cognitive mechanisms were being tested (Beran, 2015; Brucks et al., 2017). It is unclear whether the self-control measures discussed in the study might reflect a single cognitive process, or a combination of several mechanisms (Beran, 2015). Self-control has been used as a term to describe either the ability to incur a cost in order to obtain a more valuable reward instead of a less-costly, lower-value reward (Beran, 2015), or the ability to inhibit a response (MacLean et al., 2014). These two abilities are not necessarily underpinned by the same cognitive process. Work on dogs shows that even inhibition alone is not consistent across different tasks, suggesting that different tests of the same ability are not actually tapping into the same cognitive mechanisms (Brucks et al., 2017). Similarly, a recent study in pheasant chicks shows that comparisons across multiple tasks might not accurately reflect cognitive ability (van Horik and Madden, 2016). In the pheasant study, two hundred chicks experienced three foraging tasks, meant to assess whether individual variation in performance was robust and driven by real differences in cognitive ability. The study failed to find any consistent differences in problem solving ability between individuals across the three tasks, suggesting that motivation, and not cognitive capacity, was the main driver for these differences.

One way to help move past these issues would be to focus more on exploring how animals succeed at some tasks, and how they fail at others, rather than whether they simply fail or succeed at certain problems. The signature-testing approach, and research focused on cognitive processes rather than task performance, are a viable strategy for this (Kacelnik, 2009; Taylor and Gray, 2009; Seed et al., 2012; Carruthers and Fletcher, 2013; Taylor, 2014). A process-driven approach allows researchers to generate specific hypotheses about which errors, biases, limits, and specific patterns of performance identify particular cognitive mechanisms, and design experimental tasks that tease these potential processes apart. This is analogous to the strong inference approach (Platt, 1964), which aims to successively rule out alternative hypotheses through the design of experiments that specifically test these hypotheses with clear predicted outcomes for each alternative explanation. Researchers can triangulate several of these signatures within or between tasks (Heyes, 1993; Taylor, 2014), to pinpoint exactly which of several qualitative forms of intelligence different species are capable of. This approach could provide a more powerful system through which we can better address Macphail’s null hypothesis, particularly in terms of qualitative differences in intelligence. Several of the studies discussed earlier provide clear examples of behavioral signatures that constrain the possible cognitive mechanisms an animal can be using to solve a problem. For example, the presence of a distance effect bias in pigeons’ numerical discriminations, where pigeons are more accurate and quicker to discriminate number pairs when the numerical distance between them is greater, provides a clear behavioral signature that numbers are represented on an ordinal scale (Scarf et al., 2011). Similarly, the ability of crows to solve problems without mistake when downstream aspects of the problem are out-of-sight, shows they are not limited by having problems out-of-sight and so provides a clear signature for pre-planning, as decisions have to be made using mental representations of the problem (Gruber et al., 2019).

Finally, a Bayesian framework may provide useful tools in interpreting animal performances from a statistical viewpoint. Given that research questions and methods are appropriately framed, the Bayesian framework can distinguish between a lack of power in the data, and direct support for the null hypothesis (Wagenmakers, 2007; Stevens, 2017; Wagenmakers et al., 2018). In the frequentist framework, these two forms of non-significance are often confounded. This leaves researchers with inconclusive data which often ends up unpublished (the file-drawer effect; Fanelli and Fanelli, 2012; Farrar et al., 2020). In contrast, Bayesian analyses can be much more informative than their frequentist counterparts when animals fail at an experimental task. The Bayesian framework enables researchers to provide claims both for and against the existence of particular cognitive capacities in their target species, rather than it being unclear whether negatives are due to low sample size or a true failure at a task. While clearly this framework does not resolve all of the issues surrounding the interpretation of ‘evidence of absence’ in comparative psychology, it does offer a route toward bringing more quantitative and qualitative differences in intelligence to light in the literature (Stevens, 2017).

## Discussion

Macphail’s support of a null hypothesis for no quantitative differences in intelligence across species, and his claim that all intelligent behavior is association-based, fall short in the light of recent research in avian cognition. Avian cognition provides clear evidence for robust differences in intelligence among avian species, as well as between birds and other taxa, and for problem solving that extends beyond simple associative learning.

However, Macphail’s criticisms of comparative psychology are relevant to this day and can inspire researchers to make more advances in this field. Thirty-five years ago, Macphail highlighted the difficulty in establishing whether animals fail at a task because they cannot understand it, or because their performance was affected by variations in methodology (Macphail, 1982, 1985; Macphail and Bolhuis, 2001). Today, much of the field still grapples with this distinction. Researchers often cannot tell why subjects fail at some tasks but not others, and comparative psychology suffers from widespread publication bias (Farrar et al., 2020).

Macphail also highlighted that some of the preconceptions of the field at the time but had not been appropriately tested. One of these was the belief that intelligence varies predictably across species, with humans showing the greatest intelligence, followed by their closest relatives (Jensen, 1980). According to this view, one might expect an inverse correlation in intelligence with evolutionary distance from humans and other primates. Macphail argued that such a *scala naturae* assumption might be erroneous (Macphail, 1985), so helping move the field past this early anthropocentric attitude and toward the present day, where researchers focus on testing intelligence across a phylogenetically broad array of animal species, albeit often still with tests that have been designed for human intelligence.

In sum, while some of Macphail’s claims do not hold up to the current body of evidence, a number of his criticisms of the field of comparative psychology still hold in the present day. We suggest three strategies researchers can use to combat these issues: (i) using baseline tasks to ensure that contextual variables cannot explain subjects’ failure at test (Jelbert et al., 2016); (ii) focusing on a signature-testing, process-driven approach, that specifically seeks to pinpoint the cognitive mechanisms that animals rely on to solve problems (Kacelnik, 2009; Taylor and Gray, 2009; Seed et al., 2012; Carruthers and Fletcher, 2013; Taylor, 2014); and (iii) taking advantage of the Bayesian framework to distinguish between support for the null hypothesis and a lack of statistical power (Wagenmakers, 2007; Stevens, 2017; Wagenmakers et al., 2018). Put together, these three strategies can help researchers identify both quantitative and qualitative differences in intelligence between species, learn from animals’ successes as well as their failures, and triangulate evidence for complex cognition that is not rooted exclusively in associative learning.

## Author Contributions

All authors listed have made a substantial, direct and intellectual contribution to the work, and approved it for publication.

## Conflict of Interest

The authors declare that the research was conducted in the absence of any commercial or financial relationships that could be construed as a potential conflict of interest.
